# Active Microbiota of *Penaeus stylirostris* Larvae: Partially Shaped via Vertical and Horizontal Transmissions and Larval Ontogeny

**DOI:** 10.3390/microorganisms12030608

**Published:** 2024-03-19

**Authors:** Nolwenn Callac, Carolane Giraud, Dominique Pham, Dominique Ansquer, Nelly Wabete, Viviane Boulo

**Affiliations:** 1 Ifremer, IRD, Université de la Nouvelle-Calédonie, Université de La Réunion, CNRS, UMR 9220 ENTROPIE, 101 Promenade Roger Laroque, 98897 Nouméa, New Caledonia; carolane.giraud@spygen.com (C.G.); dominique.pham@ifremer.fr (D.P.); dominique.ansquer@ifremer.fr (D.A.); nelly.wabete@ifremer.fr (N.W.); viviane.boulo@ifremer.fr (V.B.); 2 Institut des Sciences Exactes et Appliquées (ISEA), University of New Caledonia, 98800 Nouméa, New Caledonia

**Keywords:** active microbiota, shrimp, larvae, taxa transmission, specific microbiota, biomarkers, shrimp ontogeny

## Abstract

During their entire lifecycle, mariculture animals are farmed in water that contains various microorganisms with which they are in close associations. Microbial exchanges between the animals and their surrounding water can occur. However, little is known about the interactions between shrimp larvae and water, and more especially, about larval bacterial selection and microbiota modulation across ontogeny. To address this gap, using HiSeq sequencing targeting the V4 region of the 16S rRNA molecule, we investigated the active prokaryotic diversity and structure of healthy *Penaeus stylirostris* larvae and seawater. Comparisons between different larval stages revealed evidence of stage-specific microbiotas and biomarkers, a core microbiota common to all stages, and shared taxa between successive stages, suggesting vertical transmission of bacterial taxa. Comparisons between stage-specific microbiotas and core microbiotas with water storages highlighted that many taxa associated with the larvae were originally present in the natural seawater, underlining horizontal transmission of bacteria from water to larvae. As some of these lineages became active at specific larval stages, we suggest that larvae were able to modulate their microbiota. This study provides insight into larvae-microbiota interactions at the larval stage scale.

## 1. Introduction

Aquatic animals are under the influence of their surrounding water and, therefore, of the water microbiota, which is involved in various biogeochemical cycles, such as carbon or nitrogen, and in the animal’s health. Some microorganisms can be pathogenic, while others may act as antipredators or probiotics [1,2,3,4,5,6,7]. Microbial exchanges can occur between water and macroorganisms’ tissues, such as the gut, gill or mantle, and aquatic animals can select specific lineages from the water to enrich their microbiota [2,8,9]. The microbial lineages associated with the host become part of the animal microbiota and form the holobiont. Therefore, the holobiont is a composite organism that encompasses the host, a *Eukaryotic* species, and the associated microbiota that provides it with more genetic and functional capabilities [10,11]. Indeed, the microbiota can play great roles in a host’s health, immunity, development or fitness [12,13]. Nowadays, it is assumed that microbial–host interactions within the holobiont are mostly driven by genetic co-evolution and ecological relationships [10,11]. Recent studies have also pointed out the importance of both biotic and abiotic interactions on host microbiota composition [14,15]. However, little is known about the processes involved in forming a holobiont and about host–microbiota interactions. Indeed, among these processes, we can cite selection pressures for microbial lineages from the same host, the concept of an “ecosystem on a leash” for animals and plants that promotes growth, and therefore, the activities of microbial lineage that bring beneficial features for host immunity and physiology, as well as overall biotic and abiotic interactions [11,16]. In penaeid–microbiota interactions, the host’s health, developmental stages, molting stages and the use of probiotics are factors known to have a strong influence on shrimp microbiota. Scarce information is available about how microbial lineages are transmitted to the host during the larval stage [1,17,18], even though vertical and horizontal transmissions have been noted from the breeders to the eggs and nauplii but not at later larval stages [19,20]. Therefore, biotic and abiotic factors influencing the microbiota associated with penaeid larvae at a given stage need further consideration, as well as host–microbiota genetic relationships. Indeed, procaryotic communities are influenced by both deterministic and stochastic processes. Deterministic processes are related to abiotic and biotic selection based on environmental conditions such as temperature, pH, nutrient resources, nature of the host habitat and biological interactions such as predation, competition and the host’s age. These factors can impact microbial structure and diversity. On the other hand, stochastic mechanisms involve ecological drift, dispersion or random change in the community (Zhou and Ning, 2017 [21] and references therein). Several studies have shown that these two processes are complementary and occur simultaneously together to shape the microbiota structure [21,22]. Recent studies have demonstrated that the shrimp (*Penaeus vannamei*) larval microbiota at its early stage was mostly influenced by stochastic processes, mainly through dispersal mechanisms [3]. It has been demonstrated that the microbiota inhabiting the gut of larval shrimps of *P. vannamei* was driven by variable selections rather than by host ontogeny [23]. The authors also showed that the succession of the prokaryotes inhabiting the larval gut was mainly driven by the age of the shrimps and that the microbiota was inherited from the earliest stages [23].

Shrimp aquaculture is one of the main sectors that provide seafood worldwide [24]. In New Caledonia, *Penaeus stylirostris*, also known as the Pacific blue shrimp, was introduced at the beginning of the 1970s. Since the 1990s, Pacific blue shrimps have been reared in a semi-intensive way, leading to a current annual production of almost 1500 tons, while the production had reached 2000 tons at the beginning of the 2000s (FAO “Fisheries and Aquaculture”). This decrease is mainly due to two seasonal epizooties involving two *Vibrio* species that cause dramatic mortalities affecting juveniles and adult shrimps grown in earthen ponds [4,25] and to larval mortality occurring at all larval stages in hatcheries for which no causes have been found yet [26,27,28]. So far, no evidence of larval septicemia has been revealed. Thus, multifactorial causes, as well as dysbiosis of the microbiota of the rearing water and/or of the larvae, have been hypothesized as factors inducing larval death. In previous studies dealing with the microbiota of the rearing water-hosting larvae with contrasted survival rates (from very low survival rates of 15% to 70%), we identified biomarkers specific to good or poor larval survival rates [27,28]. However, we lack data about the microbial diversity and structure associated with healthy *Penaeus stylirsotris* larvae. In order to fill this gap, this study aimed to (1) access the evolution of the microbial diversity and structure associated with the larvae at each developmental stage, (2) investigate specific and core microbiota that can provide evidence of putative bacterial vertical transmissions between stages as well as horizontal transmissions from the water to the larvae and (3) highlight stage-specific biomarkers of healthy larvae to later develop rearing monitoring tools. Using Illumina HiSeq sequencing targeting the V4 region of the 16S rRNA molecule of the active prokaryotes, we highlighted microbial diversity associated with each larval stage and identified ASVs that were vertically transmitted from one stage to the next one, as well as lineages horizontally transmitted from the seawater to the larvae. We also underlined stage-specific microbiota that seemed to be modulated by the host and specific biomarkers of a given larval stage.

## 2. Materials and Methods

### 2.1. Study Design, Samples Collection and Storage

This study was realized in February 2019 in the experimental shrimp hatchery of the Station Aquacole de Saint Vincent, a shrimp farming research facility (Boulouparis, New Caledonia). In the hatchery, the breeding of mature *Penaeus stylirostris* broodstock was performed by artificially inseminating females, as described previously by Pham et al. (2012) [26]. The water used to fill the hatching and rearing tanks was collected from Saint Vincent Bay after undergoing various treatments, as explained in Callac et al., 2022 and 2023 [27,28]. Briefly, lagoon seawater was pumped using a pumping device located in the Bay, which was a 1 cm-pore-size device, into a first reservoir (ResI). Then, the water circulated through both a sand filter and a 10 µm filter prior to being stored either (1) in second water storage (ResT), where the water was treated using continuous circulation through a skimmer and a sequence of filtration through 10 and 5 µm filters for 3 days before being used to fill the hatching tanks; or (2) directly into a 2 m^3^ storage container implemented with intensive bubbling (ResNT) before being used for the rearing tanks. On the insemination day (D-1), hatching tanks were filled with water from the secondary reservoir (ResT), and 5 g.m^−3^ of ethylenediaminetetraacetic acid (EDTA, Merck France, Trosly-Breuil, France) was added in all tanks, as well as intensive bubbling. EDTA is used as a metal chelator in all the hatcheries of the territory to overcome the putative toxicity of high metal concentration in the seawater (personal communication with hatcheries’ managers). In those tanks, female breeders (1 per tank) were left in the dark for a few hours to spawn before their retrieval, as described by Giraud et al., 2022 [19]. The following day, the eggs hatched to give the first larval stage: the nauplii.

The following day, on Day 0 (D0), 100 L rearing tanks were filled with water from the storage container (ResNT). Additionally, 5 g.m^−3^ of EDTA and 2 ppm of erythromycin were added, along with intensive bubbling, before the nauplii addition. Erythromycin was used under veterinary guidance for prophylactic purposes to impede larval mortalities [24,25]. Prior to their transfer to the rearing tanks, the nauplii were sorted out from egg debris and unhatched eggs, rinsed with water from the secondary reservoir (ResT), and transferred at a density of 180 larvae per liter. During the rearing period, antibiotics were added to the tanks on days 3, 5, 7 and 9 at the same concentration as on D0. Throughout the 10 days of larval rearing, no water exchange was applied. Several times per day, larvae were fed using the following protocol, as described in Callac et al., 2022 and 2023 [27,28]: microparticles were added five times per day, and frozen *Tetraselmis* sp. were given once a day to feed the larvae at the zoea 1 and 2 stages; then, from zoea 3 to post-larvae, microparticles as well as living *Artemia* sp. nauplii (between 20 to 40 nauplii per shrimp larvae per day) were given twice a day.

Lagoon seawater from the first water storage (ResI) was collected 3 days before the insemination, while seawater from the other storage containers (ResT and ResNT) was sampled on the insemination day. For each water sample, 1 L of water was filtered on 0.2 µm sterile membrane filters (S-Pak, Millipore, Burlington, MA, USA), and all filters were stored at −80 °C until RNA extractions. On the reproduction day (D-1), after spawning and females’ removal, around 100 eggs were collected using sterilized pliers and stored in sterile microtubes of 2 mL. On Day 0 (D0), just before the larvae were transferred into the rearing tanks, around 100 nauplii were collected using a 120 µm pore size net and sterilized spoon and stored in 2 mL microtubes. During the experimentation, 3 tanks were monitored, and about 100 larvae were sampled every morning in the same way as the nauplii. Similar to the filters, microtubes with eggs and larvae were kept at −80 °C until RNA extractions.

### 2.2. Quotidian Determination of Larval Survival Rates and Larval Stages

Larval survival rates were assessed daily by taking the average of 3 direct counts of the number of dead and living larvae in 100 mL samples for each rearing tank. This allowed us to define the Larval Survival Rate (LSR) for each day using the following calculation:

LSR = 100 ∗ (counted living larvae/initial number of nauplii added on D0 in each tank).

Larval stages were established by observing 30 larvae per tank using a binocular magnifying glass, as described previously in Callac et al., 2022 and 2023 [27,28]. Each day, using the modified equation of Maddox and Manzi, the calculation of the Larval Stage Index (LSI) was determined as follows:

LSI = (0 × Nii + 1 × Z1 + 2 × Z2 + 3 × Z3 + 4 × M1 + 5 × M2 + 6 × M3 + 7 × PL)/N,

where Nii, Z1, Z2, Z3, M1, M2, M3 and PL correspond, respectively, to the number of larvae observed in the nauplius, zoea 1, zoea 2, zoea 3, mysis 1, mysis 2, mysis 3 and post larvae 1 stages, and N represents the total number of observed larvae.

### 2.3. RNA Extractions, Reverse Transcriptions, Sequencing and Sequence Processing

Total RNA extractions were conducted using commercial kits and following the manufacturer’s protocols: RNAeasy Powerwater kit (Qiagen, Hilden, Germany) for the filters and RNeasy mini kit (Qiagen, Hilden, Germany) for the eggs and the larvae. The total RNAs were reverse transcripted into complementary DNA (cDNAs) as described previously in Callac et al., 2022 and 2023 [27,28]. Briefly, each reaction was conducted using RNA at 200 ng/µL, M-MLV reverse transcriptase at 200 U/μL (PROMEGA, Madison, WI, USA), 2 µL of random hexamers at 50 µM, 4 µL of Buffer 5X, 2 µL of a mix of dNTP a 10 mM each and 1 µL of Rnase/Dnase free water. The reactions were performed in a Veriti^TM^ instrument (Applied Biosystems, Waltham, MA, USA), with the following program: 10 min at 25 °C, 2 h at 42 °C and 5 min at 85 °C. The cDNAs were stored at −80 °C until shipping to MrDNA (Molecular Research LP, Shallowater, TX, USA), where the sequencing of the V4 region of the 16S rRNA molecule was performed using the universal primer set 515f-806R [29,30], according to the manufacturer’s protocol with a 2 × 150 bp paired-end run. Illumina HiSeq sequencing was conducted with an average sequencing depth of 20 k raw reads.

Prior to reading the treatment, the raw data were demultiplexed using the fastqSplitter available on the MrDNA website (https://www.Mrdnalab.com/mrdnafreesoftware/fastq-splitter.html, accessed on 20 September 2022) [20]. Then, the sequences were treated using the DADA2 [31] package, available in the Rstudio software (version 4.3.1), where we selected all the reads with a quality score (QC) above 30. As described in Callac et al., 2023 [28], the parameters used were a maxEE (maximum expected error) set at 2, a maxN (maximum N) set at 0, and a truncation based on quality scores (truncQ) set at 2. The chimeras were removed using the consensus method. Then, the Silva 138 (SSU Ref NR99 database) was used to assign the taxonomy [32]. Before downstream analysis, we removed sequences that were not affiliated with or assigned to the *Eukaryota*, *Mitochondria* or *Chloroplasts*. All the 16S rRNA data are available in the NCBI SRA repository (BioProject ID PRJNA736535, BioSamples SAMN39924754 to SAMN39924780 for all the samples except samples ResI, ResT, M4_Egg1, M4_Egg2, M4_Nii1 (collected on D0) and M4_Nii2 (collected on D0), respectively, available in SAMN19659073, SAMN19659074, SAMN19659075, SAMN19659076 and sample ResNT available in SRP324193, SAMN31027756).

### 2.4. Downstream Microbial Analysis

The alpha diversity indexes—Observed, ACE, Shannon and Inverse Simpson (InvSimpson)—were estimated using phyloseq (phyloseq package in RStudio [33]), while the Good’s coverage was calculated using RStudio. Kruskal–Wallis tests (rstatix package in Rstudio) were conducted on the alpha diversity indexes to exhibit statistically significant differences between the rearing day and the larval stage.

As conducted in previous studies, before further downstream microbial analysis, the whole ASV table was normalized using the Count Per Million (CPM) method with the edgeR package under the RStudio software (version 4.3.1) [19,20,27,28]. Beta diversity was visualized by building an NMDS based on a Bray–Curtis dissimilarity matrix using phyloseq packages [33] and ggplot2 [34] in Rstudio. The beta diversity of the whole procaryotic community composition was analyzed according to the rearing days and larval stages using PERMANOVA (non-parametric test, permutational multivariate analysis of variance) with the Adonis function in Rstudio.

LEfSe (Linear Discriminant Analysis (LDA) effect size) [35] was performed to distinguish microbial biomarkers at the genus and ASV phylogenetic level using the microbiomeMarker package [36] in RStudio, with a threshold set at 4. Then, using the microeco package [37], a correlogram based on the Spearman correlation was built between the genera identified in the LEfSe and the larval stages to ensure biomarker identification for each larval stage.

Before constructing the Venn diagram to highlight a core microbiome among the larvae through their larval development, along with specific microbiotas, we defined 5 groups of larvae according to their stage: egg, nauplii D0 (reared in water from the ResT), nauplii D1 (reared in the same water as the larvae, e.g., water from ResNT), zoea and mysis. Then, to untangle the role of the seawater on the larval during the rearing, both core and specific microbiotas were compared to the microbiotas of the water storage. Venn diagrams were built using the Jvenn web application tool [38] (http://bioinfo.genotoul.fr/jvenn/example.html, accessed on 7 November 2023).

Core microbiota from the Venn diagram was used to infer putative microbial functions in larvae according to their stage, using FRAPROTAX: Functional Annotation of Prokaryotic Taxa [39] in the microeco package [37]. As described in Colette et al., 2023 [40], FAPROTAX is a tool that predicts metabolic phenotypes and ecological features and functions based on published data [39,41].

## 3. Results

### 3.1. Zootechnical Parameters

**Figure 1:**
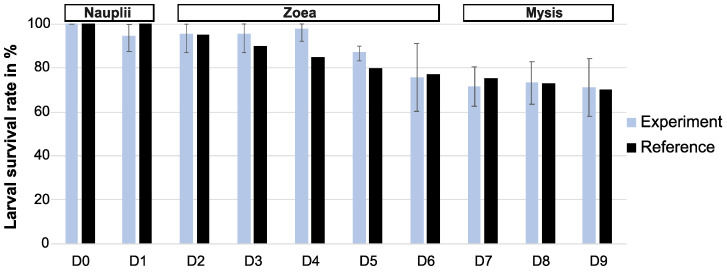
Mean percent of larval survival in the 3 tanks through the 10 days of the rearing, shown in grey, compared to the reference in black. The reference corresponds to the larval survival and stages obtained for a given day. For each day, the reference was calculated using data from 10 years of successful larval rearing (Ifremer data, personal communication with Pham).

The larval survival rates (LSRs) were globally higher than the reference during the first days of rearing (from D0 to D5, except on D1, which could be due to inaccurate counts during larval observations) or comparable to the reference from D6 to D9 (Figure 1 and Table S1). The lowest larval survival rate was on the last day of the experiment (D9), with a larval survival rate value of 71%. Larval observations showed that all the nauplii had metamorphosed to reach the zoea stage on day 2 (D2) and that all the zoea had transformed into mysis on day 7 (D7) (Table S1). The larvae transitioned into post-larvae on D9–D10.

### 3.2. Dynamic of the Active Microbial Diversity Associated with the Larvae

To characterize the active procaryotic diversity associated with the larvae and the water storages, a metabarcoding approach was used. After removal of the reads affiliated with the *Eukaryota*, *Mitochondria* and *Chloroplasts*, as well as the unassigned sequences, a total of 11,739,853 sequences distributed into 6448 ASVs were obtained from the HiSeq sequencing. The ASVs span into 21 phyla, 2 within *Archaea* and 28 within *Bacteria*. The smallest library was composed of 117,085 reads and corresponded to the sample L_D7_B (larvae collected on D7 from tank B), while the largest library, consisting of 552,990 reads, corresponded to the sample L_D9_C (larvae collected on D9 from tank C). The Good’s coverage, calculated using the whole ASV table, revealed an overall average above 99.85% (Table S1), indicating that the sequencing depth was sufficient.

**Figure 2:**
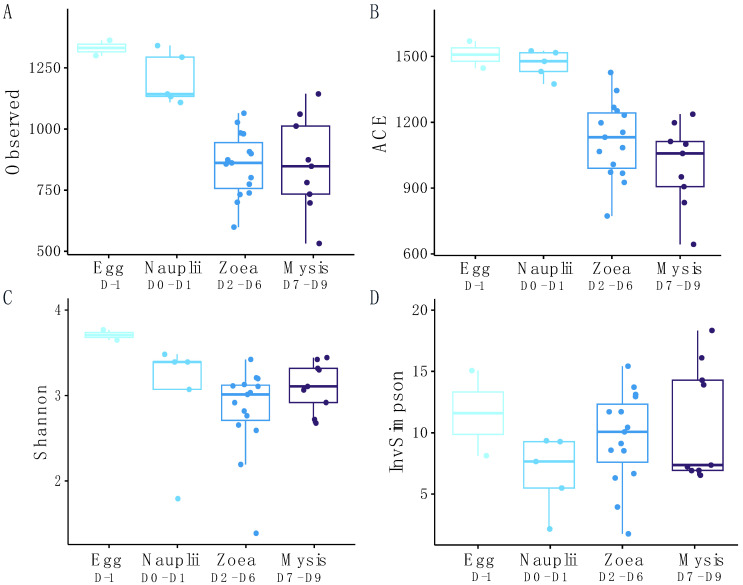
Alpha diversity indexes of the microbial diversity associated with the larvae. (**A**) Observed, (**B**) ACE, (**C**) Shannon and (**D**) Inverse Simpson. Turquoise stand for the egg samples, light blue for the nauplii, medium blue for the zoea and navy blue for the mysis samples. Data are available in Table S1.

The alpha diversity values, shown in Figure 2 and Table S1, exhibit that the richness estimated using the Observed and ACE indexes globally decreased as the larvae metamorphosed. The microbial richness of the water storage was in the same range as that of the eggs and nauplii. Estimated with both Shannon and Inverse Simpson indexes, the evenness displayed a similar trend to the richness indexes, with higher values at the egg and nauplius stages as well as in the reservoirs. Kruskal–Wallis tests, followed by Dunn tests performed when needed (when significance was observed after Kruskal–Wallis) on the alpha diversity indexes. They exhibited that the Observed and ACE indexes were significantly different, with *p*-value < 0.05, and were influenced by the stage, especially between the egg and the zoea, the egg and the mysis, the nauplii and the zoea, and the nauplii and the mysis (Table 1). Kruskal–Wallis tests, followed by Dunn tests when needed, conducted on the alpha diversity indexes according to the rearing day, revealed that the rearing day significantly impacted the richness indexes (Observed and ACE indexes, with *p*-values both at 0.012). The pairwise comparisons indicated that D-1 was significantly different from D2, D3, D5, D6, D7 and D8 (see the pairwise comparison in Table S2). No difference was observed in evenness indexes regarding the larval stage or the rearing day.

**Figure 3:**
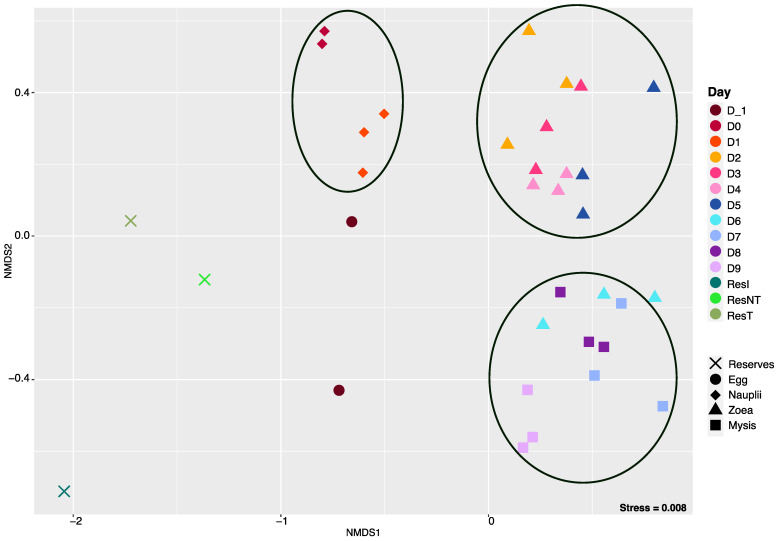
Ordination of the microbial diversity associated with the larvae.

The entire normalized ASV table was used to calculate beta diversity using the Bray–Curtis dissimilarity matrix, and sample clusterization was visualized on an NMDS with a stress of 0.008 (Figure 3). The ordination displayed three main clusters. The first one gathered the nauplii samples from D0 and D1. The second grouped the zoea samples collected from D2 to D5. The last cluster encompassed the last samples of zoea (zoea collected on D6) and all the mysis samples (from D7 to D9). Apart from these clusters, the egg and the reservoir samples were separated. The clustering was mostly consistent with the larval stages, except for the zoea on day 6, suggesting a transition phase between the zoea and the mysis stage. To confirm the clusters and the NMDS repartition and evaluate the influence of the larval stage and the rearing day, a PERMANOVA was performed. The PERMANOVA displayed that the day and the stage explained 80% of the variability among the samples (both *p*-values at 0.001), while 20% were not described by these factors. The factor that accounted for the most in the microbiota variability was the larval stage, explaining 49% of the microbial diversity variability.

The nonmetric multidimensional scaling (NMDS) method and a Bray–Curtis dissimilarity matrix exhibit microbial clustering linked to the larval stage, with stress at 0.008. Each sampling day corresponds to a specific color in the figure, as displayed in the description on the right side of the NMDS, and each symbol corresponds to a larval stage or to water storage. ResI corresponds to the primary reservoir sample, and ResT and ResNT, respectively, stand for the storage reservoir with water treated via continuous circulation through a skimmer and filter (ResT) and water stored directly in the 2 m^3^ storage container (ResNT).

**Figure 4:**
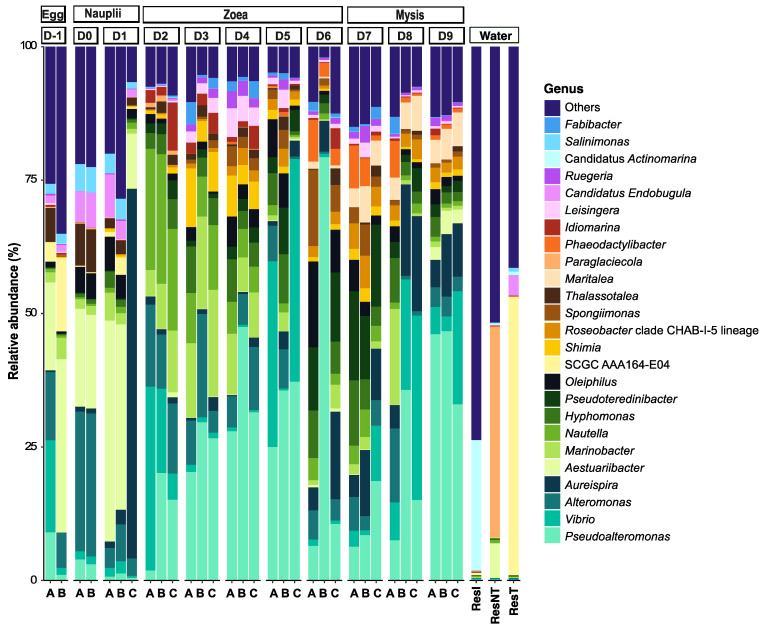
Microbial composition of the larvae throughout the rearing period and of the water storage.

The active microbiotas of the three water reservoirs were clearly different from each other, suggesting that filtration had an effect on microbial diversity. Also, most of their lineages belonged to genera that were not displayed in Figure 4, as they did not represent more than 1% of the total abundance of the reads in the other samples and were not among the 25 most abundant genera. Among the taxa present in at least 1% of the total abundance in all samples, the Candidatus *Actinomarina* were highly present in the lagoon seawater, while *Paraglaciecola* dominated in ResNT and SCGC AAA164-E04 in ResT.

The egg samples (D-1) were mostly composed of members of the *Vibrio*, *Alteromonas*, *Aestuaribacter*, *Thalassotaleae* and SCGC AAA164-E04 genera. The nauplii samples (D0 and D1) were quite similar in terms of main taxa but not regarding their prevalence, even though their rearing waters were different between the 2 days. The nauplii on D0 were reared with water from ResT, and the nauplii on D1 were stored in tanks with water from ResNT. They mainly contained taxa affiliated with the *Aestuaribacter*, *Alteromonas*, *Thalassotalea*, *Oleiphilus*, Candidatus *Actinomarina* and *Salimonas* genera and *Aureispira* for the sample D1 tank C. A shift in microbial diversity occurred between D1 and D2. Then, the microbial genera that prevailed from day 2 to day 5 were *Vibrio*, *Nautella*, *Marinobacter*, *Alteromonas*, *Shimia*, *Idiomarina* and *Leisingera*, along with an increase in *Pseudoalteromonas* abundance. On day 6, the main taxa were related to *Pseudoalteromonas* (especially in tank B), *Aureispira*, *Pseudoterenibacter*, *Oleiphilus*, *Spongiimonas* and *Phaeodactylibacter* genera. From day 7 to day 9, the microbial diversity associated with the larvae was mostly made of *Pseudoalteromonas*, with an increasing abundance of *Pseudoterenibacter* and a decreasing abundance of *Aureispira*, *Maritalea* and *Hyphomonas*.

Relative abundances of the main prokaryotic genera are displayed as a percentage of the total microbial sequences per sample. Only the 25 most abundant genera are displayed on the barplot; the other genera that were not among the 25 most abundant genera were pooled in the “Others” category. Water stands for water storage: ResI corresponds to the primary reservoir sample, ResT to the secondary reservoir sample, and ResNT to the storage reservoir.

### 3.3. Specific and Core Microbiotas Associated with the Larval Stages and the Reservoirs

**Figure 5:**
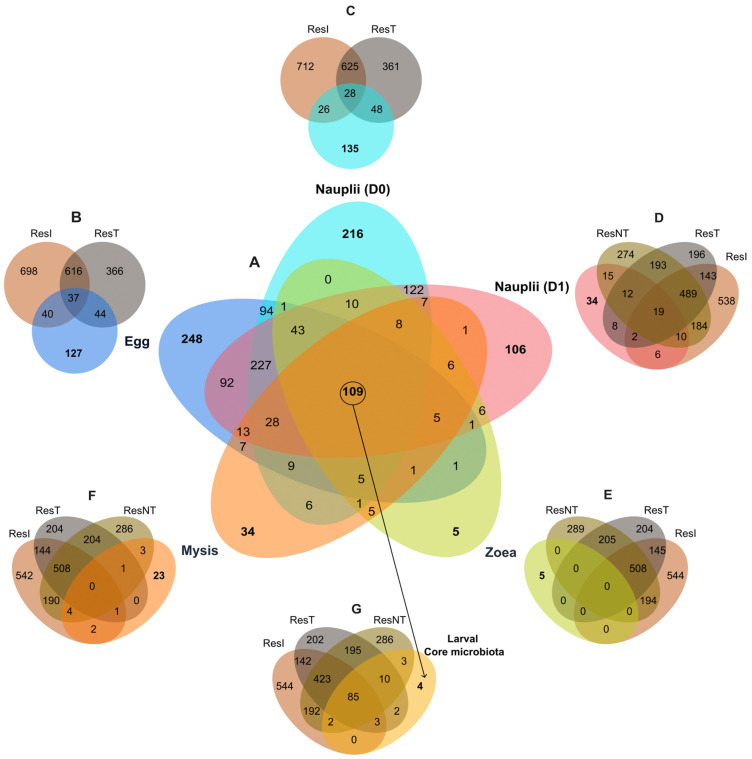
Specific and core microbiotas of the larvae according to their larval stages and comparison with the water storages. (**A**) Venn diagram of commons ASVs among all the larvae samples; (**B**,**C**) Venn diagrams of commons ASVs between the water storages ResI and ResT, and (**B**) the eggs and (**C**) the nauplii collected on D0, (**D**–**F**) Venn diagrams of commons ASVs between the specific ASVs of the water storages ResI, ResT and ResNT, and (**D**) the nauplii collected on D1, (**E**) the zoea, (**F**) the mysis; (**G**) Venn diagram of the core microbiota of the larvae and the ASVs of the water storages. Colored ellipses are related to specific ASVs, in blue = eggs, turquoise = nauplii collected on D0, light red = nauplii collected on D1, light green = zoea, orange = mysis, light orange = larval core microbiota, brown ellipse = primary reservoir ResI, grey = secondary reservoir ResT and khaki = the storage reservoir ResNT. The core microbiota, made of 109 ASVs shared by all the samples, is represented by the overlapping of all the ellipses. Numbers inside the ellipses and in the overlapping represent the number of ASVs of a given condition.

To determine specific ASVs of a given larval stage and shared microbiotas, seven Venn diagrams were built. The first one was constructed to compare the microbiota of the five defined larval groups: egg reared in the water from ResT, nauplii D0 also reared with water from ResT, nauplii D1 reared with water from ResNT, zoea and mysis also reared with water from ResNT (Figure 5A). The diagram displayed that the egg had 248 specific ASVs, 216 for the nauplii D0, 106 for the nauplii D1, only 5 for the zoea and 34 for the mysis. All the larval stages co-owned a core microbiote made of 109 ASVs (Figure 5A). When compared together, the egg and the nauplii samples (D0 and D1) shared 407 common ASVs, while the nauplii and the zoea co-owned 170 ASVs, and the zoea and the mysis gathered 140 common ASVs (Figure 5A and Figure S1). As the larvae were reared using water from different storages, with the egg and nauplii on D0 reared with water from ResT and the nauplii on D1 up to the mysis with water from ResNT, the core microbiota and the stage-specific microbiotas were compared to the microbiota of the three water storages. These three water storages were kept in comparison with the nauplii D1, zoea, mysis and core microbiota. Indeed, as the eggs and nauplii on D0 were reared in the water from ResT, lineages could have been attached or enclosed with the larvae and not yet active at a given stage. The comparison between the eggs and the water storages ResI and ResT highlighted that the eggs shared 121 ASVs with the seawater from ResI and ResT and harbored 127 specific ASVs (Figure 5B). The nauplii collected on D0 exhibited 135 specific ASVs and co-owned 102 ASVs with the water storages ResI and ResT (Figure 5C), while the nauplii collected on D1 shared 72 ASVs with the seawater (ResI, ResT and ResNT) and owned 34 specific ASVs (Figure 5D). No common ASV was found between the microbiota of the zoea and the three water storages, while the zoea owned only five specific ASVs (Figure 5E). The mysis harbored 23 specific ASVs and shared 11 ASVs with the three water storages (Figure 5F). The comparison between the core microbiota of the larvae and the water storages displayed that only 4 ASVs were specific to the core microbiota owned by the larvae, while the other 105 ASVs were common with the reservoirs, and most of these ASVs were present in the first water storage (ResI) (Figure 5G). Together, the Venn diagrams underlined the important role of the water storage microbiotas on the larval microbiota, as numerous ASVs identified in the water storages (ResI, ResT and ResNT) were also detected several times in the larval microbiota.

### 3.4. Biomarkers Associated with the Larval Stages

Figure 2 highlights a partitioning of the microbial diversity of a given larval stage. In order to investigate the differentially abundant biomarkers associated with the larval stages, we made two LEfSe analyses. The first LEfSe was conducted to examine the ASVs that were statistically more abundant according to the larval stage (Figure S2) and to see if these ASVs were previously found in water storages (ResI, ResT and ResNT). With a threshold set at four (Figure S2), the analysis showed that seven biomarkers were specific to the eggs, six were prevalent at the nauplii stage, five at the zoea stage and seven at the mysis stage. Interestingly, biomarkers related to the *Pseudoalteromonas* genus were statistically enriched at the egg stage (1 biomarker), at the zoea stage (1 biomarker), and at the mysis stage (2 biomarkers) (Figure S2). The same trend was observed for the biomarkers affiliated with the *Marinobacter*, with two detected at the egg stage and one at the zoea stage (Figure S2). Biomarkers related to the *Auresipra* genus were detected once in the nauplii and once at the mysis stage. The other biomarkers were affiliated with unique genera such as SCGC AAA164-E04 for the egg, Candidatus *Endobugula* for the nauplii, *Hyphomonas* for the zoea and *Pseudoterenibacter* for the mysis (Figure S2). By comparison with the water storages, it appeared that all of these ASVs statistically enriched in a given stage were already present in all the water storages except ASV11, which was not detected in ResI nor in ResNT.

**Figure 6:**
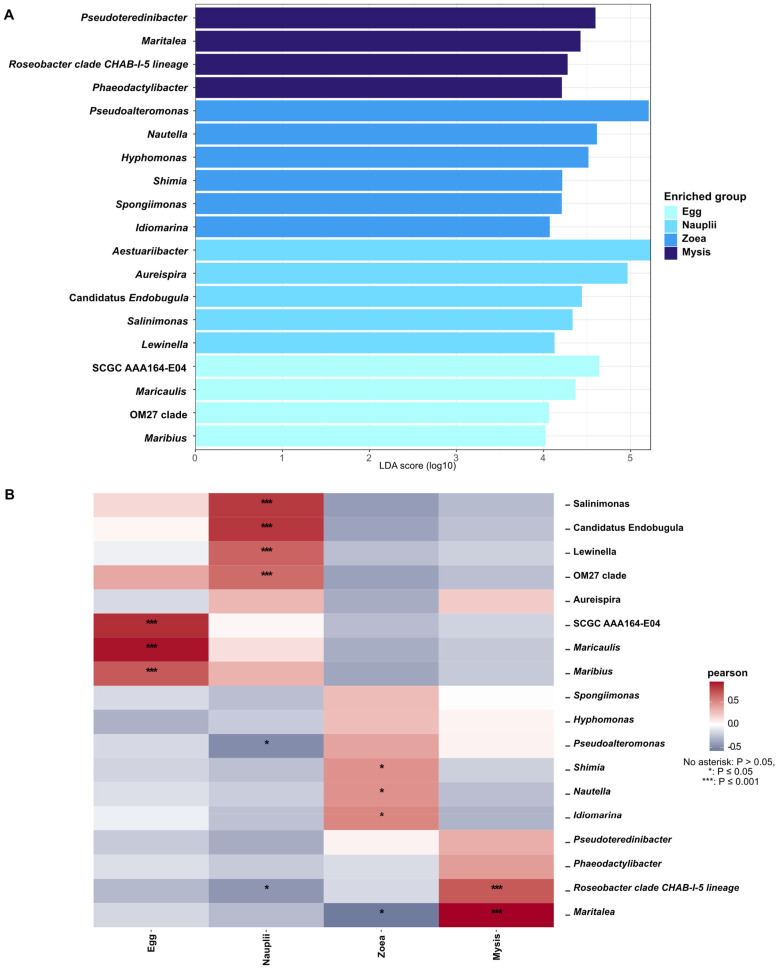
Differentially abundant genera according to the larval stage. (**A**) LEfSe displaying the genera which were statistically more abundant according to each larval stage. (**B**) Correlogram of the biomarkers detected via the LEfSe according to the larval stage. Heatmap color gradient is linked to Spearman correlation coefficient intensity: red stands for positive correlation, while blue corresponds to negative correlation. Significant correlations are noted with an asterisk (*), with no asterisk: *p* > 0.05, *: *p* ≤ 0.05, ***: *p* ≤ 0.001.

Then, in order to untangle which genus was specifically enriched at a specific stage for larval health monitoring purposes, a second LEfSe analysis was conducted (Figure 6A). The analysis revealed that four genera were enriched in the eggs: SCGC AAA164-E04, *Maricaulis*, OM27 clade and *Maribius*. The *Aestuariibacter*, *Aureispira*, Candidatus *Endobugula*, *Salinimonas* and *Lewinella* were biomarkers of the nauplii. The specific biomarkers of the zoea were *Pseudoalteromonas*, *Nautella*, *Hyphomonas*, *Shimia*, *Spongiimonas* and *Idiomarina*. Four biomarkers were statically enriched in the mysis: *Pseudoterenibacter*, *Maritelea*, *Roseobacter* Clade CHAB-I-5 lineage and *Phaeodactylibacter.* Thereafter, to determine which biomarker was indeed associated with a given stage with the aim to emphasize specific proxies for healthy larval monitoring, a Pearson correlation was performed using the biomarkers highlighted by the LEfSe at the genus level (Figure 6B). The correlogram exhibited that *Maricaulis* was greatly positively associated with the eggs as well as SCGC AAA164-E04 and *Maribius*. The correlogram also displayed that *Salinimonas* and Candidatus *Endobugula* were highly positively correlated with the nauplii and, to a lesser proportion, that the nauplii were also associated positively with *Lewinella* and OM 27 Clade. Weak positive correlations were found between the zoea and the genera *Shimia*, *Nautella* and *Idiomarina*. *Maritalea* was highly positively correlated with the mysis, as well as the *Roseobacter* Clade CHAB-I-5 lineage. The correlations support the biomarkers detected with the LEfSe, except for the OM27 clade.

### 3.5. Putative Functions Associated with the Genera of the Specific and Core Microbiota of the Larvae

**Figure 7:**
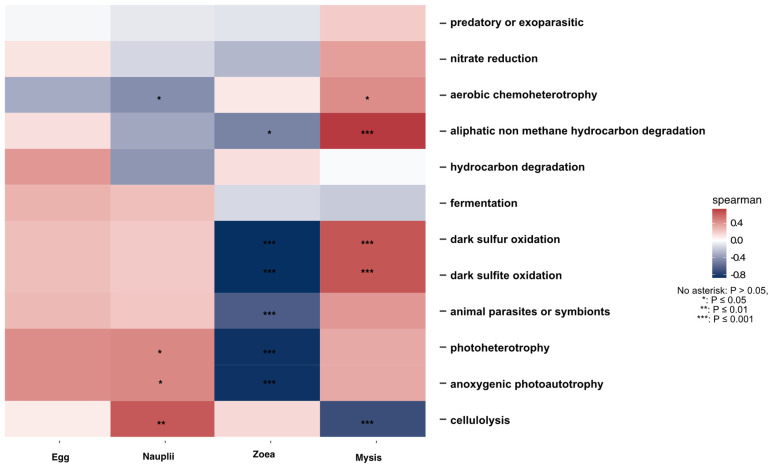
Correlogram of putative ecological functions assigned with FAPROTAX to the core microbiotas of all the larvae. The heatmap color gradient related to Spearman correlation coefficient intensity: red for positive and blue for negative correlations. Significant correlations are denoted with an asterisk (*), no asterisk: *p* > 0.05, *: *p* ≤ 0.05, **: *p* ≤ 0.01, ***: *p* ≤ 0.001.

The putative functions associated with the genera of the specific and core microbiotas were determined using FRAPROTAX (Figures S3 and S4). Spearman correlations were conducted on the putative functions associated with the core microbiota according to each larval stage (Figure 7). The eggs did not exhibit significant positive correlations with putative functions of the core microbiota, while the nauplii were positively correlated with the cellulolysis function and weakly with the anoxygenic photoautotrophy and photoheterotrophy (Figure 7). The putative functions of the core microbiota associated with the zoea stage exhibited strong negative correlations with four functions: anoxygenic photoautotrophy, photoheterotrophy, dark sulfide oxidation and dark sulfur oxidation. The mysis showed a great positive correlation with aliphatic nonmethane hydrocarbon degradation and, to a lesser extent, with dark sulfur oxidation, as well as with dark sulfite oxidation, while a strong negative correlation was underlined with the cellulolysis function (Figure 7).

## 4. Discussion

Our study aimed to explore the evolution of the larval microbiota according to the larval stage, investigate the specific and core microbiota of each larval stage, untangle the role of the water storages in establishing the larval microbiota, provide evidence of vertical transmission and detect stage-specific biomarkers of healthy larvae. To reach these objectives, we assessed the active microbial diversity associated with the larvae and the water storages by extracting total RNAs from the collected samples, followed by reverse transcriptions into cDNAs. RNA was chosen over DNA, as its short turnover allows it to monitor recent populations and living assemblages in an ecosystem and perform biological surveys [42,43,44,45].

A clear dynamic of the active microbial diversity associated with the larvae was evidenced throughout the whole rearing (Figure 3 and Figure 4), as previously shown for the microbial diversity of other penaeids shrimp larvae: *Penaeus indicus* [46] or *P. vannamei* [3,47,48]. Strikingly different microbial compositions were observed between D1 (nauplii) and D2 (zoea), between D5 (zoea) and D7 (mysis) with a transition on D6 (zoea), along with the three distinct clusters underlined by the NMDS (Figure 3), regrouping larvae microbiota samples according to the larval stage. Clusters were confirmed with the PERMANOVA, which indicated that 49% of the variability among the samples was influenced by the larval stage. These shifts in the active microbial community, occurring at each important larval metamorphosis to reach a superior stage, highly suggest that the larvae might modulate their microbiota, as shown by Wang et al., 2020 for *P. vannamei* larvae [3,47]. Along with the PERMANOVA exhibiting the stage effect on the larval microbiota, the decrease in the richness alpha diversity indexes through the larval stages (Figure 2 and Table S1) might indicate that, at each stage, the larvae have performed a microbial selection, especially between the nauplii and the zoea stages. The same pattern was observed in *P. vannamei* larvae across their life stages, with a drop in the number of lineages between the nauplii and the zoea [3]. Together, this suggests a host selection of microbial taxa throughout larval ontogenesis. Thus, it is not surprising to observe a richness reduction occurring greatly after the nauplii stage, just after the mouth opening. After this stage, the nutritional mode drastically changed for the larvae, going from vitellotroph feeding on their own yolk reserve at the nauplii stage to microparticles and microalgae feeding, as well as predation of nauplii of *Artemia* sp. at the zoea and mysis stages [3,49]. Thus, the lack of differences in the microbial composition between the eggs and the nauplii (Figure 4 and *p*-value > 0.05 for all the alpha diversity indexes in Table 1) might rely on their food status and on the absence of an open digestif tract in interaction with the surrounding rearing water. The same observation can be made for the active microbiota associated with the zoea and mysis, where no statistical differences were shown between the alpha diversity indexes (Table 1). Similarly, other studies have noticed that the composition of the shrimp larval microbiota became more homogeneous at the zoea stage, which could be due to the host selection of specific lineages with specific functions [3,47]. The assignation of putative functions of the specific larval stage microbiotas with FRAPROTAX exhibited different profiles according to the stage, with even a marked difference between the nauplii collected on day 0 and on day 1, which corroborates that the host selects specific microbial lineages with specific functions (Figure S2). Even if the profile of the ecological functions of the specific microbiota was stage-specific, common functions such as anaerobic chemoheterotrophy and aerobic heterotrophy were found at every stage. All specific microbiotas encompassed the function “human pathogen septicemia”, and others had “plant pathogen”. This does not mean that such pathogens were part of the larval microbiota. Indeed, FRAPROTAX uses data from cultivation experiments to identify microbial functions, metabolic activities or ecological roles [39,41]. Therefore, if all the cultivated members of the same taxa used for the database perform a given function, this function will be attributed to all cultivated and uncultivated lineages related to that taxa [41]. In the same way, all *Vibrio* species are not human pathogens.

Another proof that the larvae might select and modulate their active microbiota is shown in the Venn diagram, which displays, for all larval stages, that the larvae had their own specific and unique microbiota alongside a common core microbiota (Figure 5). This was also shown by Giraud et al., 2021 and 2022, who found that the eggs and the nauplii of *P. stylirostris* larvae had both a specific microbiota and a core microbiota [19,20]. In their studies, these authors also proved that several lineages were shared between the eggs and the nauplii, highly suggesting a potential vertical transmission of specific microbiota [19,20]. The same trend is observed in this study, where direct successive stages shared common lineages, such as the egg and nauplii samples that co-owned 407 active taxa, the nauplii and zoea 170, and the zoea and mysis 140. In addition, 152 common ASVs were co-owned by all stages, from the eggs to the zoea, and a core microbiota of 109 ASV highlights greatly that specific ASVs were retained and transmitted through vertical transmission during the whole larval cycle (Figure 5A and Figure S1). Vertical transmission of specific bacterial taxa from parents to their offspring has also been demonstrated in *Rimicaris exoculta*, a shrimp species living in hydrothermal vent areas [50,51], as well as in other marine hydrothermal or cold seep organisms such as clams [52] and aquatic macroorganisms such as fish or sponges [53,54]. Among the core microbiota, several lineages such as *Alteromonas*, *Ascidiaceihabitans* (formerly *Roseobacter*), *Halomonas*, *Litoricola*, *Leisingera*, *Micrococcus*, *Pseudoaltermonas*, *Rhodovulum*, *Ruegeria* and *Sulfitobacter* might exhibit probiotic or beneficial activities [55,56,57,58,59,60,61,62,63,64]. Others have a putative antimicrobial activity or ixotrophic activity with evidence of ASV related to *Aureispira* or the OM27 Clade (*Bdellovibrionaceae,* affiliated with the Bdellovibrio and like organisms) [6,65]. Other taxa from the core microbiota were previously characterized as biomarkers in the rearing water according to the health status of *P. stylirostris* larvae, with *Fabibacter* or *Marinobacter* identified as biomarkers of healthy zoea, *Tenacibaculum* as a biomarker of healthy mysis, *Nautella* as a proxy of both healthy zoea and mysis, *Idiomarina* enriched in healthy zoea and unhealthy mysis, *Aestuariicoccus* enriched in unhealthy zoea and mysis, or *Marivita* as a biomarker of unhealthy mysis [28]. Finding common biomarkers in healthy and unhealthy larvae reinforces our hypothesis concerning microbial dysbiosis causing larval mass mortalities. Since core ASVs were already present in the eggs and might have been kept throughout the whole life cycle, we can hypothesize that the larvae and maybe even the breeders have selected particular bacterial communities harboring specific functions to be beneficial for the animal’s health and welfare. Indeed, members of the core microbiota might act as probiotics or be involved in immune homeostasis [66,67]. The putative functions and ecological activities of the core microbiota assigned with FRAPROTAX displayed different abundance profiles according to the larval stage (Figure S4), while the correlogram indicated specific putative functions among the core microbiota according to the larval stage (Figure 7). This might indicate that the core microbiota, by varying bacterial abundance across the larval stage, aimed to maintain specific needed microbial functions for the holobiont and to reinforce others according to the stage.

Owing to the dynamic nature of microbial diversity associated with the larvae during the rearing, we identified stage-specific biomarkers that highlight genera statistically enriched at a given larval developmental stage using both LEfSe and Pearson correlation (Figure 6A,B). The specific biomarkers of the egg stage were the *Maricaulis*, SCGC AAA164-E04 and *Maribius*. Members of the *Maricaulis* genus are generally oligotrophs, with members able to degrade hydrocarbons, and are known to inhabit marine environments with poor nutrient availability [68], suggesting that they were probably present on the surface of the eggs, which contain yolk rich in proteins and lipids [69,70]. A similar suggestion can be made for members of the *Maribus* genus, as they belong to the *Rhodobacteraceae* family and encompass marine oligotroph microorganisms, some of which are involved in quorum sensing [71,72]. Scarce information is available about SCGC AAA164-E04; apart from that, they belong to the *Verrucomicrobiota* phylum and were detected in the water of an oxygen-depleted basin of the Gulf of California, Mexico [73]. Two biomarkers highly related to the nauplius stage are the *Salinimonas* and Candidatus *Endobugula* genera. Species related to the *Salimonas* genus are marine organisms isolated from marine sediments and the tube of deep-sea hydrothermal polychaetes, exhibiting the ability to degrade polysaccharides and aromatic hydrocarbons [74,75,76,77]. Candidatus *Endobugula* is a well-known bacterial symbiont of the Bryozoan *Bugula neritina*, and some strains are able to produce bryostatins, bioactive polyketides that are believed to act as antipredator chemical defenses for the bryozoan host larvae [78,79]. We can thus hypothesize that Candidatus *Endobugula* might have an activity in nauplii defenses against putative predators or pathogens. While members of *Salimonas* might be involved in food acquisition via macromolecule degradation. Surprisingly, the zoea did not have strong positive correlations with their biomarkers detected via LEfSe: *Shimia*, *Nautella* and *Idiomarina*. The genus *Shimia* has been found in the gut of *P. vannamei*, and this genus is correlated with detoxification genes [80,81]. In the juvenile *Totoaba macdonaldi*, *Shimia* has been reported to be beneficial for the fish intestine by enhancing the absorption of nutrients and improving fish growth [82]. The genus *Nautella* as a biomarker is controversial, as it has been detected as a biomarker in both unhealthy larvae of *P. vannamei* and in the water used for their rearing [1,83]. However, other studies underlined this genus as a biomarker of healthy *P. stylirostris* larvae in rearing water [28] or of healthy *P. vannamei* larvae and shrimps [47,84]. In this study, members of the *Nautella* genus were related to healthy larvae. *Idiomarina* are generally found in marine environments, saline settings or hydrocarbon-contaminated areas and are able to degrade amino acids to produce exopolysaccharides and biosurfactants [85,86,87]. Members of *Idiomarina* were also recorded in symbiotic association with the mussel *Mactra stultorum*, where they are involved in crude oil degradation [88]. Their association with the zoea might indicate a role in food acquisition for the larvae. *Maritalea* and *Roseobacter* Clade CHAB-I-5 lineages were mysis biomarkers. Members of the *Maritalea* genus are marine organisms isolated from ciliates, red algae and sediments trapped in plastic remains in marine areas [89,90,91]. *Maritalea* were also found in symbiotic association with the nudibranch *Rostanga alisae*, where this genus is involved in trophic interaction via the transfer of fatty acids to the host [92]. Members of the *Roseobacter* Clade CHAB-I-5 lineage remain uncultivated, yet the annotation of their metagenomes exhibits genes involved in a photoheterotrophy lifestyle, sulfur oxidation as well as interesting features such as bacterium–bacterium interactions or bacterial–host interaction. Indeed, genes involved in quorum sensing (*luxRI* gene) and in the type VI secretion system (T6SS) that allows the injection of antibacterial toxin to competitors or pathogens were detected in their genomes [93,94]. Thus, evidence of the *Roseobacter* Clade CHAB-I-5 lineage as a biomarker of the mysis might indicate their role in larval protection against putative pathogens. These identified genera as stage-specific biomarkers of healthy larvae might be further used as a bio-surveillance tool for monitoring farmed shrimp. Changes in biomarker detection might then indicate an upcoming larval disease and mortality episode, as we have already shown with biomarkers of rearing water inhabiting larvae shrimp [27].

The active microbiota associated with the larvae also seemed to be driven by the microbiota of the water storages (ResI and ResT) used during the early larval stages (egg, nauplii on D0 and D1). Indeed, as displayed in Figure 5, many taxa detected in the specific microbiota of a given larval stage were originally detected in the natural seawater (ResI, ResT and ResNT) used for egg-hatching and nauplii rearing before transfer into the rearing tanks. Antibiotics were added to the rearing tanks on Day 0 (before larval transfer), D3, D5, D7 and D9, and might have affected the microbiota of the rearing water by exerting selective pressure on sensitive taxa [95]. In a previous article dealing with the rearing water microbiota, we had evidence that the addition of erythromycin to the rearing water, before the transfer of the nauplii into the rearing tanks, had only affected the rare biosphere, with less than 3% of the active microbiota being specific to the rearing water with or without antibiotics [27]. We can, therefore, emphasize that without antibiotic addition in the rearing water, more taxa would probably have been shared between a given larval stage and the water storage. The biomarkers identified at the ASV level exhibited the same pattern, as all of them were also detected in the water storages, which showed the great role of the natural seawater (lagoon) microbiota in both the larvae and the larval microbiota establishment. This highly suggests that horizontal transmission of bacterial taxa and/or host selection of specific taxa seem to occur from the water to the larvae. Such microbial transmission between the water storages and the eggs and nauplii of *P. stylirostris* was previously shown by Giraud et al., 2021 and 2022 [19,20], and microbial exchanges between the water and the animal tissues (gut and gills), as well as host selection of specific microorganisms, has also been shown [8,9,96]. Moreover, many active lineages of the water storages ResI and ResT were detected in the later developmental stages (e.g., zoea or mysis), which might underline that these lineages became active according to the larval development stage. In shrimps, shifts and evolution of the microbial diversity, as well as microbial functions, have been demonstrated to be linked to host ontogeny, physiology or food [3,17,97,98]. These data also highlight that the egg and nauplii had recruited and selected prokaryotes from the rearing water that would later be part of their active microbiota. Thus, complex interactions between the holobiont and the water microbiota seem to occur in the early larval stages, participating to partially shape the host microbiota.

## 5. Conclusions

Taken together, our data and results reveal that the active microbial diversity associated with the *Penaeus stylirostris* larvae was dynamic across the larval development, with evidence of both specific lineages of a given larval stage along with a core microbiota common to all stages. Larval ontogeny seems to be partially involved in the microbiota evolution, as major shifts occurred at metamorphosis to reach a superior stage. Our data also suggest a host selection of microbial taxa across larval development, along with the implication of vertical transmission of specific lineages from stage to stage. The holobiont seems to be influenced by the lagoon water microbiota that drove at least a part of the active microbiota associated with the larvae through horizontal transmission. The host–microbiota appears, then, to be shaped via both vertical and horizontal transmission of bacterial lineages, by shrimp ontogeny, and by complex interactions with the natural and probably with the rearing water. Further studies to investigate the host–microbiota–rearing water interactions at each stage through meta-transcriptomic approaches will allow us to untangle the role of ontogeny from that of the rearing water on active microbiota establishment and dynamic. In this study, we have also managed to highlight stage-specific biomarkers of healthy larvae that could later be used as biosurveillance tools to monitor shrimp farming.

## Figures and Tables

**Table 1:** Pairwise comparison (Dunn test) on the alpha diversity indexes showing a significant *p*-value after the Kruskal–Wallis test, according to the larval stage. Significant differences (*p* < 0.05) are indicated in bold.

Compared Groups		Observed	ACE
Egg	Nauplii	0.631	0.829
Egg	Zoea	**0.009**	0.026
Egg	Mysis	**0.015**	**0.007**
Nauplii	Zoea	**0.002**	**0.004**
Nauplii	Mysis	**0.007**	**0.001**
Zoea	Mysis	0.882	0.297

## Data Availability

The raw HiSeq Illumina sequencing data are available on the NCBI SRA repository (BioProject ID PRJNA736535; BioSamples SAMN39924754 to SAMN39924780 include all the samples except for samples ResI, ResT, M4_Egg1, M4_Egg2, M4_Nii1 (collected on D0) and M4_Nii2 (collected on D0), which are available in SAMN19659073, SAMN19659074, SAMN19659075, SAMN19659076, respectively; sample ResNT is available in SRP324193, SAMN31027756). Metadata about the larval stage and survival are available in Table S1.

## Supplementary Materials

The following supporting information can be downloaded at https://www.mdpi.com/article/10.3390/microorganisms12030608/s1, Figure S1: Differentially abundant ASVs according to the larval stage, Figure S2: Relative abundance of the main putative ecological functions assigned with FAPROTAX to the specific microbiota of each larvae stage, Figure S3: Relative abundance of the main putative ecological functions assigned with FAPROTAX to the core microbiota common to all larvae stages; Figure S4: Relative abundance of the main putative ecological functions assigned with FAPROTAX to the core microbiota common to all larvae stages; Table S1: Samples characterization and Alpha diversity indexes, Table S2: Pairwise comparison (Dunn test) on the alpha diversity indexes according to the rearing day.
