# Supplementary material for: Active Microbiota of Penaeus stylirostris Larvae: Partially Shaped via Vertical and Horizontal Transmissions and Larval Ontogeny
Source: Microorganisms. 2024 Mar 19;12(3):608. doi: 10.3390/microorganisms12030608 (PMC10976216; doi:10.3390/microorganisms12030608)
Supplement: Supplementary file 1 [file microorganisms-12-00608-s001.zip › microorganisms-2900702-supplementary.pdf]

Figure S1. Specific and core microbiotas of the larvae according to their larval stages.
A) Venn diagram of shared ASVs among the eggs and the nauplii collected on D0 and on D1. B) Venn diagram of shared ASVs among the nauplii collected on D0, on D1 and the zoea. C) Venn diagram of shared ASVs between the zoea and the mysis.
Colored ellipses correspond to group-specific ASVs of the larvae in: blue = egg, turquoise = nauplii collected on D0, light red = nauplii collected on D1, light green = zoea, orange = mysis. The overlapping area between all the ellipses, corresponds to the core microbiome. The numbers inside the ellipses and in the overlapping zones correspond to the total number of ASVs present in a given condition.

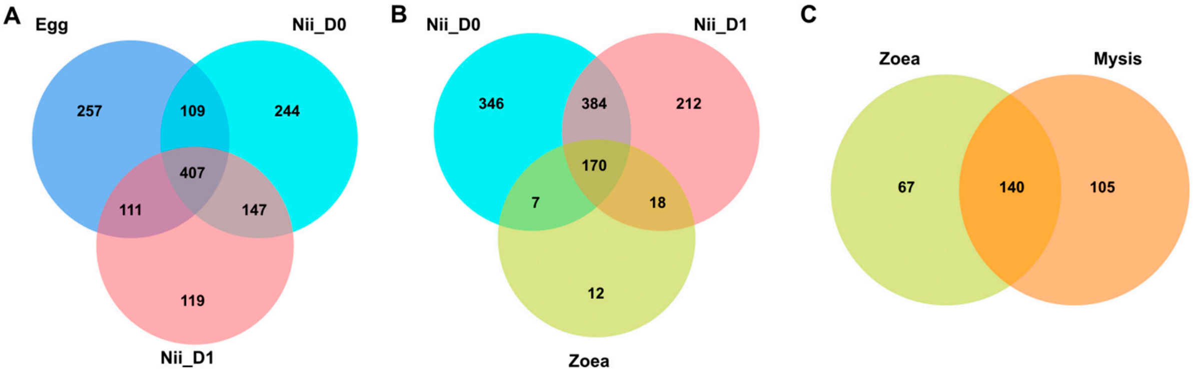

Figure S2. Differentially abundant ASV according to the larval stage.
LEfSe, linear discriminant analysis (LDA) effect size, exhibiting the ASVs significantly more abundant in the larvae according the larval stage.

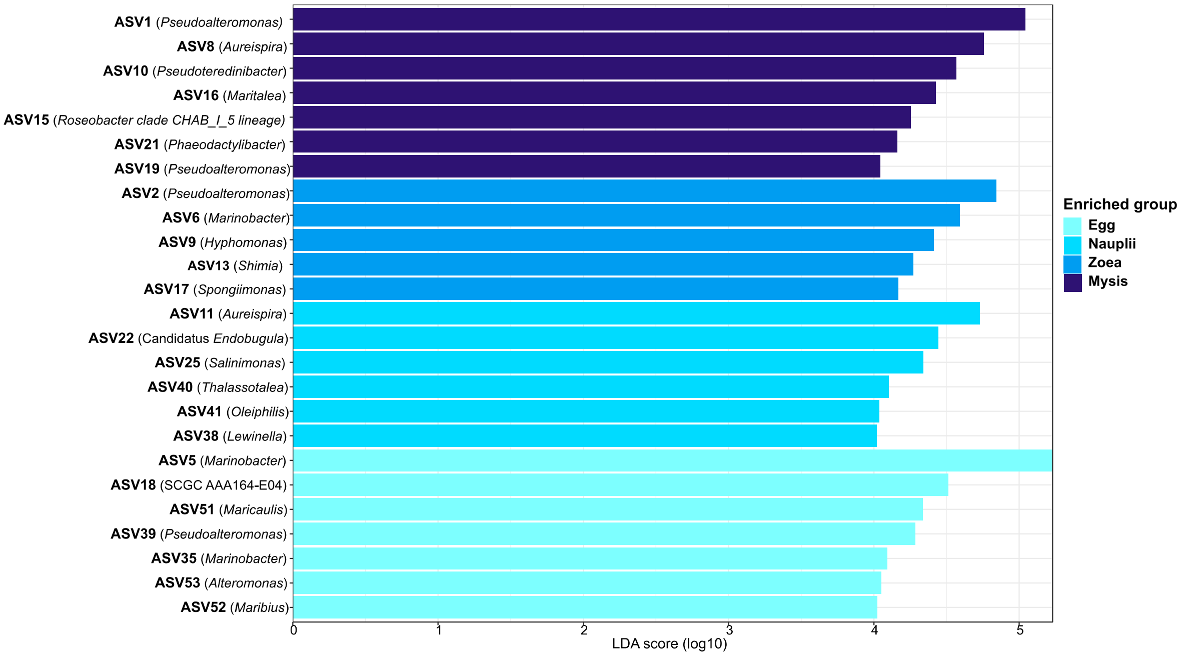

Figure S3. Relative abundance of the main putative ecological functions assigned with FAPROTAX to the specific microbiota of each larvae stage.

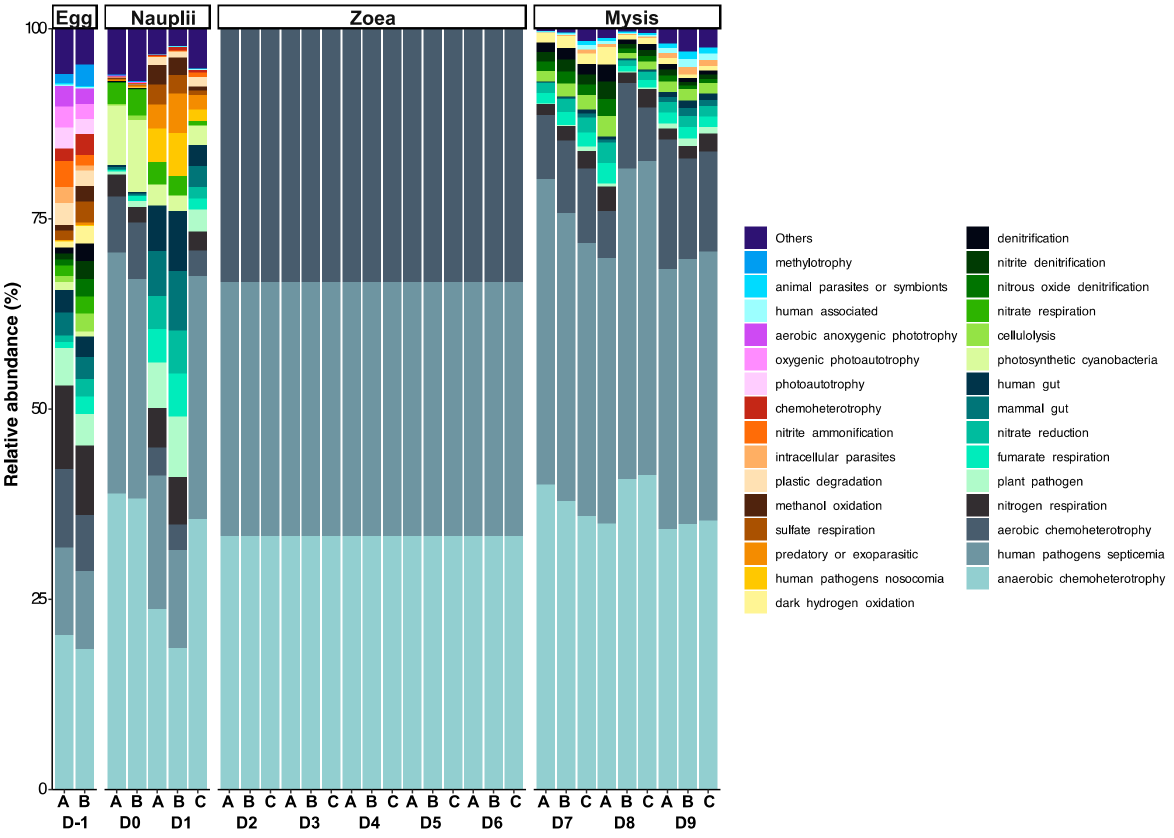

Figure S4. Relative abundance of the main putative ecological functions assigned with FAPROTAX to the core microbiota common to all larvae stages.

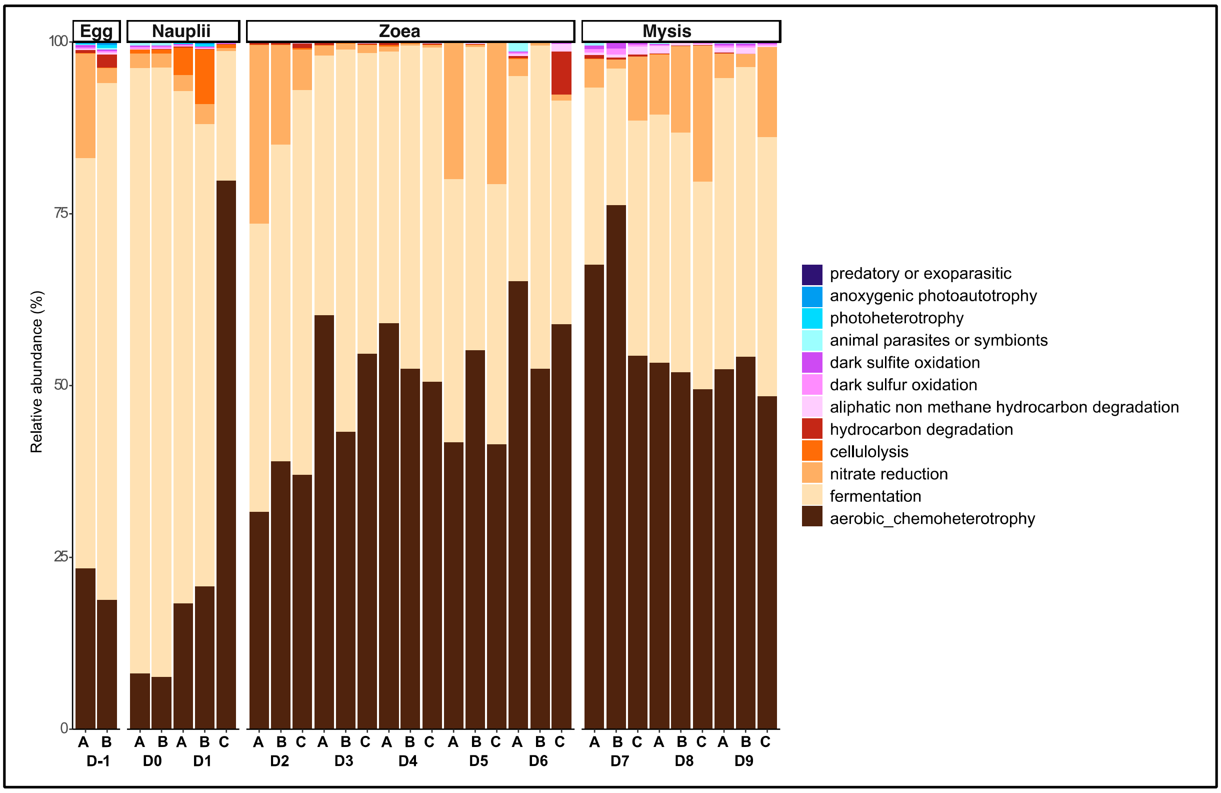

Table S1. Samples characterization and Alpha diversity indexes.
NA stands for non-applicable and PL for post-larvae

Sample	Rearing *day*	Survial rate in %	Larval stage	Observed	ACE	Shannon	Inverse Simpson	good's coverage
Egg1	D_1	100	Egg	1300	1569.64	3.77	15.06	99.85
Egg2	D_1	100	Egg	1363	1446.68	3.65	8.13	99.93
Nii1_D0	D0	100	Nauplii	1143	1431.41	3.39	9.35	99.90
Nii2_D0	D0	100	Nauplii	1109	1374.37	3.39	9.28	99.90
L_D1_A	D1	100	Nauplii	1294	1517.01	3.07	5.49	99.94
L_D1_B	D1	92.4	Nauplii	1341	1525.12	3.48	7.66	99.94
L_D1_C	D1	90.7	Nauplii	1133	1477.66	1.79	2.15	99.93
L_D2_A	D2	100	Zoea	770	1084.31	2.59	6.67	99.94
L_D2_B	D2	89.8	Zoea	728	1008.24	2.76	8.54	99.92
L_D2_C	D2	96.9	Zoea	1023	1426.85	3.13	11.72	99.93
L_D3_A	D3	96.3	Zoea	895	1232.34	3.11	13.13	99.94
L_D3_B	D3	92.2	Zoea	696	972.50	2.82	9.13	99.92
L_D3_C	D3	98	Zoea	976	1344.52	3.03	10.44	99.92
L_D4_A	D4	87	Zoea	980	1267.76	3.01	10.08	99.94
L_D4_B	D4	96.7	Zoea	870	1197.58	2.92	8.58	99.91
L_D4_C	D4	109.1	Zoea	903	1154.27	3.21	13.73	99.94
L_D5_A	D5	82.6	Zoea	857	1131.91	2.66	6.32	99.92
L_D5_B	D5	87.4	Zoea	853	1066.89	3.11	11.72	99.94
L_D5_C	D5	91.1	Zoea	594	772.98	2.19	3.94	99.93
L_D6_A	D6	72.6	Zoea	797	926.16	3.20	12.95	99.94
L_D6_B	D6	73.3	Zoea	734	967.68	1.39	1.75	99.96
L_D6_C	D6	80.7	Zoea	1060	1252.09	3.42	15.42	99.95
L_D7_A	D7	78.1	Mysis	734	906.73	3.42	16.10	99.90
L_D7_B	D7	69.6	Mysis	532	643.99	3.44	18.33	99.89
L_D7_C	D7	66.5	Mysis	698	834.17	3.32	14.29	99.91
L_D8_A	D8	73.3	Mysis	848	1100.48	3.30	13.91	99.90
L_D8_B	D8	72.8	Mysis	781	951.02	2.72	6.94	99.93
L_D8_C	D8	73.3	Mysis	874	1057.82	2.68	6.54	99.95
L_D9_A	D9	75.9	Mysis-PL	1061	1197.76	3.11	6.92	99.96
L_D9_B	D9	70.9	Mysis-PL	1012	1112.43	3.07	7.23	99.96
L_D9_C	D8	66.5	Mysis-PL	1144	1236.76	2.92	7.37	99.97
ResI	NA	NA	NA	1391	1509.31	3.90	16.54	99.88
ResNT	NA	NA	NA	1196	1513.62	2.74	5.22	99.92

ResT	NA	NA	NA	1062	1312.84	3.13	6.24	99.89

Table S2. Pairwise comparison (Dunn test) on the alpha diversity indexes according to the rearing day.

In bold, values indicate significant differences (P < 0.05).

Compared groups		**Observed**	**ACE**
D_1	D0	0,547	0,616
D_1	D1	0,769	0,971
D_1	D2	**0,023**	0,092
D_1	D3	**0,030**	0,124
D_1	D4	0,092	0,164
D_1	D5	**0,011**	**0,017**
D_1	D6	**0,035**	**0,028**
D_1	D7	**0,002**	**0,002**
D_1	D8	**0,025**	**0,019**
D_1	D9	0,359	0,133
D0	D1	0,714	0,557
D0	D2	0,107	0,256
D0	D3	0,133	0,322
D0	D4	0,305	0,399
D0	D5	0,061	0,067
D0	D6	0,147	0,099
D0	D7	**0,015**	**0,010**
D0	D8	0,115	0,072
D0	D9	0,797	0,340
D1	D2	**0,027**	0,054
D1	D3	**0,037**	0,078
D1	D4	0,119	0,110
D1	D5	**0,012**	**0,007**
D1	D6	**0,042**	**0,012**
D1	D7	**0,002**	**0,000**
D1	D8	**0,030**	**0,008**
D1	D9	0,486	0,085
D2	D3	0,902	0,870
D2	D4	0,512	0,743
D2	D5	0,774	0,436
D2	D6	0,854	0,566
D2	D7	0,356	0,110
D2	D8	0,967	0,461

D2	D9	0,129	0,838
D3	D4	0,594	0,870
D3	D5	0,682	0,346
D3	D6	0,951	0,461
D3	D7	0,296	0,078
D3	D8	0,935	0,367
D3	D9	0,163	0,967
D4	D5	0,346	0,268
D4	D6	0,637	0,367
D4	D7	0,114	0,054
D4	D8	0,539	0,286
D4	D9	0,389	0,902
D5	D6	0,637	0,838
D5	D7	0,525	0,412
D5	D8	0,743	0,967
D5	D9	0,071	0,325
D6	D7	0,268	0,305
D6	D8	0,886	0,870
D6	D9	0,183	0,436
D7	D8	0,335	0,389
D7	D9	**0,015**	0,071
D8	D9	0,140	0,346
